# Limitations and Opportunities in Open Laryngeal Organ Preservation Surgery: Current Role of OPHLs

**DOI:** 10.3389/fonc.2019.00408

**Published:** 2019-05-22

**Authors:** Giovanni Succo, Erika Crosetti

**Affiliations:** ^1^Oncology Department, University of Turin, Turin, Italy; ^2^Head Neck Oncology Unit, Candiolo Cancer Institute, FPO IRCCS, Turin, Italy

**Keywords:** laryngeal cancer, partial laryngectomy, supracricoid laryngectomy, supratracheal laryngectomy, salvage surgery, quality of life, outcome, functional results

## Abstract

The current trend for treatment of intermediate-early advanced laryngeal cancer is essentially oriented toward preservation of organ and laryngeal function, and with a good potential for treating the disease. This goal can be achieved by adopting open laryngeal organ preservation surgery (OLOPS), at present mainly represented by open partial horizontal laryngectomies (OPHLs). An approach using rigorous selection criteria based on both the general condition of the patient and the local and regional extent of the disease gives excellent oncological and functional results in untreated patients. Similar outcomes, albeit slightly worse, are also obtainable in radio-recurrent and laser-recurrent patients. Troublesome postoperative management and the inconsistency of functional recovery are the main limitations for extensive application of this therapeutic strategy. The future direction is represented by simplification of the indications identifying iso-prognostic sub-categories within the T-stage, wider consensus on rehabilitation protocols, hybrid approaches to the larynx, and open minimally invasive access.

## Introduction

In recent years, the introduction of chemoradiation-based organ-sparing protocols (1–3) has demonstrated that larynx preservation is feasible even when this organ is affected by intermediate-locally advanced cancer. This approach, together with associated surgical procedures, has led to a considerable change in the therapeutic approach to laryngeal cancer, characterized by anatomical sparing of the larynx or preservation of its functions (4, 5).

Open laryngeal organ preservation surgery (OLOPS) may be applicable as a strategy to spare laryngeal function, preventing permanent tracheostomy (6) with its associated negative aspects. Despite being very rich in techniques and solutions historically, OLOPS has developed over the past 30 years within the field of open partial horizontal laryngectomies (OPHLs) (4, 7) since transoral laser microsurgery (TLM) has now universally replaced vertical partial laryngectomies (5).

OPHL has been used extensively as a surgical option in early (I–II) and intermediate stages (III). The excellent reliability of the technique is demonstrated by robust and repeatable oncological outcomes at a similar level to function sparing results (8, 9) as long as strict eligibility criteria are applied. Thanks to this rigorous approach, with a further comparative balance between pre- and post-treatment staging, OPHLs provide excellent oncological and functional results as rescue procedures in radio-recurrent (10) and laser-recurrent (11) laryngeal cancer (12). The limitations of OPHLs, procedures not currently universally adopted, lie in the fact that postoperative management is often difficult and residual laryngeal function can vary greatly among centers (12); in particular, the quality of vocal recovery can vary immensely, even in the hands of experienced surgeons.

A mini-review of recent literature, focusing on OPHLs, is reported in order to assess the changes which are occurring in open laryngeal organ preservation surgery, based on selection of patients, indications, functional outcomes, strategies, and technique refinements.

## Historic Landmarks

In the late 20th century, OLOPS has progressively been adopted as a treatment modality for laryngeal cancer at different stages: early (frequently), intermediate (occasionally, but progressively increasing), and rarely in selected advanced cases.

In 1947, Alonso introduced the concept of OPHL when describing supraglottic laryngectomy (13), and Bocca et al. (14) contributed to the widespread diffusion of the procedure, also expanding its indications.

The logic of a horizontal resection, also applied to glottic and subglottic tumors, was shared by Mayer and Rider in Vienna (15) and became established through more frequent application to intermediate stage tumors (16–20). These interventions (21), called “supracricoid partial laryngectomies” (SCPL), have gradually reached consensus in the treatment of more locally advanced laryngeal cancers (most T3). Supratracheal partial laryngectomy (STPL) was first described by Serafini (18). Supracricoid laryngectomy with tracheocricohyoidoepiglottopexy, described in 1996 by Laccourreye et al. (22) as a SCPL extended to the cricoid ring, paved the way for the development of “functional” STPL, which was described by Rizzotto et al. in 2006 (19, 23).

In 2014, the European Laryngological Society introduced a new classification “of the more common procedures according to the extent of resection, including three types of OPHL: type I—supraglottic, type II—supracricoid, and type III—supratracheal” (7). This simple classification facilitated the learning of these interventions by less experienced surgeons, and the comparison of results between different surgeons and institutions.

## Selection of Patients

The preoperative selection of patients is one of the most crucial points in obtaining a good functional outcome. Old age, an important factor when considering a patient for surgery in current practice, is no longer taken to be an exclusion criterion (24) due to the increasing divergence between biological age and chronological age. The current cut-off is shifting to 70 years, with the major focus on biological age. In fact, as demonstrated by Benito et al. using a prediction model, the increased age (>70 years) together with the widening of the intervention and resection of one arytenoid correlates with a greater probability of severe impairment of swallowing function, thus causing severe aspiration (25). Gallo et al. recently reported similar results when applying a prediction nomogram for dysphagia to 535 patients (26). Simple clinical parameters, coupled with the intermediate degree of hemodynamic stress associated with OLOPS, allow the best candidates to be identified for this procedure: the ability to climb two flights of stairs (= 4 METs) (27) as well a Karnofsky index cut-off placed at a level >80, the latter corresponding to normal activity with effort (28). Finally, the focus should be on the patient's related exclusion issues (9) as well as on the presence of serious metabolic diseases (e.g., diabetes mellitus), diseases greatly reducing lung function, diseases of the nervous system reducing expectoration and/or the ability to swallow, or severe heart disease (29).

## OPHLs in Untreated Patients

In oncological terms, the most significant feature of OLOPS, represented today essentially by OPHLs, is related to resection of the laryngeal segment affected by the disease with all its anatomical constituents: mucosal layer, membranous layer, submucosal visceral spaces, muscles and corresponding cartilage. Furthermore, the three major categories of OPHL (type I–III) can simply be combined and modulated with posterior enlargement (arytenoid or crico-arytenoid unit) or vertically upward or downward, making them suitable for the management of most laryngeal cancers.

OPHLs are today emerging as the preferred treatment for laryngeal cancer in the early and intermediate stages (rarely T1, T2, and selected T3), and even less anecdotal is the demonstration of their effectiveness in selected “early” T4a disease (4, 30–34).

After a complete resection, the prognostic outcomes of type I OPHLs, which are certainly the most consolidated ones (more than 60 years after systematic adoption), appear to be comparable to those obtained by TLM (35, 36). Despite similar results in terms of survival and laryngeal preservation (37) [5-year disease-specific survival (DSS) 72 vs. 80%, 5-year laryngeal preservation rate 80 vs. 86%], comparative analysis of laryngeal function (35) has revealed that TLM has a smaller effect on swallowing capacity than open neck supraglottic laryngectomy, resulting in lower morbidity and faster recovery.

Treatment of supraglottic cancers experience less loco-regional control than glottic tumors, since they are usually diagnosed at an advanced stage because of a higher incidence of cervical lymph node metastasis (38–42). Even considering the good results obtained by transoral approaches [TLM and transoral robotic surgery (TORS)] (43, 44), type I and type IIb OPHLs allow good outcomes in function preservation management of early-intermediate supraglottic cancer (95% at 5 years) (45). These procedures continue to be indicated in patients with difficult transoral exposure and in the case of bulky tumors.

National Comprehensive Cancer Network guidelines (33) for glottic/supraglottic cancer amenable to larynx preservation include the option of partial laryngectomy for early laryngeal cancer and only for selected T3. The definition of “selected” identifies patients who are fit for an open neck surgical procedure, where OPHL is supposed to be the exclusive treatment (T2–T3 N0 tumors with anterior commissure or transglottic spread). Very gradually, OPHLs have been adopted as an extreme larynx-sparing option in naïve T4a cases, with initial extra-laryngeal extension (9, 32).

A problem hindering the standardization of indications on the basis of the T category is the excessive heterogeneity of the cT2–cT3 and cT4 categories, both supraglottic and glottic. In fact, less experienced surgeons often find it difficult to understand why, always within the same stage of T, an OPHL is more suitable for some sub-categories of cancer rather than others.

A study on 489 type II–III OPHLs was recently carried out to identify iso-prognostic subcategories in cT3 to cT4a supraglottic/glottic cancers, and also describing their different patterns of spreading, and the modality of endo/extra laryngeal recurrence (46). Based on the anatomical compartmentalization of the larynx matched with a functional parameter (arytenoid mobility), this study allowed identification of the sub-categories in which the indication for OPHL is oncologically sound. In fact, anterior cT3 tumors (tumors without involvement of the posterior paraglottic space and normal arytenoid mobility) can be managed by OPHL; the same concepts and approach could also be adopted in the treatment of cT4aN0 with a minimal anterior extralaryngeal extension. Succo et al. reported that, despite promising results, the OPHL approach should be considered under investigation in posterior cT3 tumors due to clinical and biological behavior similar to cT4a tumors (46). This, however, results in a resident-based method useful to overcome the difficulties of indications arising from the TNM classifications; a similar approach has recently been described for the management of laryngeal cancer by TLM (47).

In patients with early and intermediate T-stage (T2-T3) treated by type II OPHLs, 5-year local control rates are above 90%, and disease-free survival (DFS) is between 70 and 90% (8, 8, 9, 9–21, 21–30, 30, 31, 31–40, 40–54). The overall survival (OS) as pooled mean is about 79.7% and the total laryngectomy completion rate due to aspiration pneumonia is low (1–3%) (6, 8, 9, 25).

In 1972, Serafini (18) described a type of OPHL called tracheohyoidoepiglottopexy to manage laryngeal cancer extending to a subglottic site by preserving the apex of the epiglottis and its subsequent pexy with the hyoid bone and the first tracheal rings. The functional results were poor due to the removal of both arytenoids compelling that author to abandon this procedure.

In the 1990s, Laccourreye et al. (23) reported an enlargement of conventional SCPL to the cricoid ring in the case of glottic tumors extending anteriorly to the subglottis. Finally, in 2006, Rizzotto et al. described the current technique of STPL (OPHL type III), allowing outcomes to be obtained which were functionally and oncologically sound (19).

The reported oncological and functional outcomes are still insufficient to adopt unquestionably type III OPHL (19, 22, 23, 55–59). However, these results are certainly interesting and need further confirmation in larger series; patients undergoing OPHL type IIa and type IIIa show “comparable long-term functional outcomes” (58) and therefore OPHL type IIIa, representing an additional option to treat glottic tumors with subglottic extension, can be considered to be a valid surgical option among OLOPS.

In patients affected by intermediate cancer (pT3), the locoregional control (LRC) and DFS at 5 years reach 88.7 and 86.4%, respectively, while in patients with an early extra-laryngeal extension (pT4a), LRC drops to 64.8% and DFS to 52.7% (22, 23).

A desirable role for a type III OPHL could be that of an enlargement procedure of a type II OPHL when resection margins are proved to be insufficient, without shifting immediately to a total laryngectomy (Table 1).

**Table 1:** Three to five year local control (LC) rates in treatment-naive patients.

**Author**	**Publication year**	**T stage**	**Type OPHL**	**No. of Pts**	**3-year LC (%)**	**5-year LC (%)**
Adamopoulos	1997		I	92	92.4	
Bocca	1983		I	407		86.5
Succo	1999		I	142		78
Spriano	1997	1-2	I–IIb	66		95.5
Maurizi	1999		I	132		74
Prades	2005		I	110	90.3	
Succo (part A)	2016	2	II–III	216		97.5
Succo (part B)	2016	3-4a	II–III	555		90.6
Chevalier	1997	2-3	II	112		94.6
Mercante	2013	3	II	32	96.2	96.2
Lima	2006	3-4	II	43		85
Laccourreye	1996	2-4	IIIa	21		88.9
Dufour	2004	3	IIa–IIb	118	93.5	91.4
Rizzotto	2015	3-4a	IIIa–IIIb	115		69.6
Schwaab	2001	1-2-3-4	IIb	146		95.8
Gallo	2005	1-2-3-4	IIa–IIb	253		91.3
Laudadio	2006	1b−2-3-4	IIa–IIb	206		93.2
Laccourreye	1998	3–4	IIb	60		98.3

## Functional Outcomes

When analyzing outcomes of OLOPS, great attention must be given to functional outcomes since any treatment has a negative impact on different functions: breathing, swallowing, voice and quality of life (QoL) (59, 60). These functional outcomes must be brought to the patient's attention when discussing the different treatment options.

The mean length of hospital stay, removal of the nasogastric tube, and decannulation times are highly variable, ranging between 5–104 days (61, 62), 10–88 days (63, 64), and 8–105 days (61, 65), respectively; however, lower variability is reported in decannulation rates (85.7–100%) (66, 67), testifying to the good patency of the neoglottis following OPHL.

Swallowing functional outcomes are generally good after OPHL, although not overlapping in the different series; however, it is even more important to determine the reasons for the extreme differences in the adopted rehabilitation protocols. During the early postoperative period, aspiration rates are higher ranging from 30 to 100% (67, 68); dysphagia is more frequent for liquids rather than solids and spontaneous recovery is observed within 6 months in 15–80.4% of cases (25, 69), and a free diet regime is achieved in the first postoperative year in the majority of patients (53–100%) (70, 71). In the longer term, most patients report occasional well-tolerated episodes of aspiration while aspiration pneumonia occurs in 0–21.7% of cases (70, 72). High-grade dysphagia and frequent aspiration pneumonia clearly affect the physical and emotional condition of patients.

Voice impairment has been recognized as the real Achilles' heel in patients undergoing OPHL type II–III, significantly compromising the emotional balance. Schindler et al. report that, in OPHL type II and III patients, the voice is produced by a neoglottis that is inherently patent at rest and in turn demonstrates substantially less volitionally induced valuing activity and resistance to airflow during voicing (71); a significant loss of air during phonation requires an increase in expiratory pressure and strength in closure of the neoglottis to achieve rigidity and improve vibration, thus producing a strained, deep and asexualized voice, which is difficult to modulate and to raise; speech is composed of short sentences, because patients are short of breath (73). Schindler et al. (12) state that maximum phonation time (MPT) implies adequacy of air support for speech and is quite low in OPHL type II–III patients, probably due to the lower resistance of the neoglottis with consequent air loss during phonation (67); thus, in order to compensate for the air wastage during phonation, the SCPL–STPL patient needs to increase neoglottal resistance and subglottic pressure with consequent vocal fatigue because of the increased physiological effort required to phonate. Interestingly, MPT appears not to be significantly affected by arytenoid removal, suggesting well-tolerated recovery of glottal closure after removal of the ipsilateral arytenoid and reconstruction of the neoglottis (12, 74).

Aiming to improve the sphincteric action of the larynx after OPHL a surgical strategy based on injection laryngoplasty with different materials can be effectively adopted in rehabilitation of dysphagia and dysphonia (75).

Few reports have focused on post-OPHL QoL and data are contradictory. QoL is a condition which is strongly influenced by psycho-social, ethnic and cultural factors. When swallowing-related QoL was analyzed, data indicate that dysphagia has only a limited impact on everyday life. Moreover, it should be noted that the vast majority of patients take food without restrictions (62, 65).

Schindler and colleagues still state that “since the voice is mainly used for everyday verbal communication, it is possible that vocal QoL is perceived by the patient as not being significantly compromised, even though the voice *per se* is rather poor” (71, 76).

## Ophl as Salvage Surgery

In recent years, the increasing attention given not only to tumor control but also to preservation of functionality has resulted in a gradual replacement of up-front total laryngectomy (TL) by radiation (RT), chemoradiation (CRT), TLM and OPHLs (2, 55, 77, 78).

Local relapse after RT and TLM is rare for T1 (range 5–13%) while for T2 laryngeal cancer, it ranges from 25 to 40% (79, 80). Steiner et al. have observed that the pattern of recurrence often demonstrates aggressive behavior arising in a field where lymphatic drainage is unpredictable and is associated with poor control rates (81). Diagnosis of recurrence is made difficult because of radiation sequelae such as edema or the frequent submucosal spreading after TLM determining a reduction in sensitivity/specificity of conventional diagnostic tools.

Several studies in the last decade have focused on the potentiality of OPHLs in terms of local control and functional results after RT and TLM (10, 11). Some studies have confirmed the feasibility of the technique, showing that the recurrence occurs in more advanced stage (82–91).

Local control at 24 months ranges between 70 and 95% (83–86, 88–90), DFS at 36 months between 70 and 90% (83–87, 89), while OS at 5 years stands between 70 and 90% (83–86, 92).

Sometimes a salvage or completion TL is required. The organ sparing rate is 85.2%, while the mean decannulation rate is 92.1% in over 200 patients reported in the literature. Laryngeal stenosis is a redoubtable side-effect (3.9% of cases) making it difficult to remove the tracheostomy. An efficient swallowing ability is achievable in almost 90% of cases. A gastrostomy dependence rate in 3.5% and aspiration pneumonia in 6.4% were reported in a cohort of 221 patients (83–86).

Data relating to phonatory outcomes after salvage OPHL are rarely reported in the literature. Pellini et al. reported an “acceptable quality of voice for most patients” (82), very perturbed and hoarse in the majority of patients with maximum phonation time (MPT) ranging from 3 to 18 s (mean, 8.3 s). Similar results were also reported by several other authors (83, 85, 87) (Table 2).

**Table 2:** Three to five year local control (LC) rates in pre-treated patients.

**Author**	**Publication year**	**T stage**	**Type OPHL**	**No. of Pts**	**3-year LC (%)**	**5-year LC (%)**
Pellini	2008	1-2-3-4	IIa–IIb	78		94.9
Spriano	2002	1-2	IIa–IIb	15		100
Deganello	2008	2-3	IIa–IIb	31		75 (LRC)
De Vincentiis	2015	1-2-3-4	IIb	68	79 OS	
Laccourreye	1996	1-2-3	IIa–IIb	12	83.3	
Makeieff	2005	1-2	IIa–IIb	23		74 66.6 (with organ preservation)
Luna-Ortiz	2009	1-2-3	IIa	40	87	
Paleri (review)	2011		IIa–IIb + vertical	560	86.9 (2 years) (84–89.5)	

*LRC, locoregional control; OS, overall survival*.

## Technical Improvements

Although there is a consensus on standard surgical techniques for OPHLs, the advent of robotic surgery has opened a window on the possibility of performing this surgery using hybrid approaches combining a limited transcervical approach and TORS (93, 94). The hybrid approaches, offering direct visualization during tumor resection before laryngotomy and full closure of the laryngopharynx defect, might facilitate postoperative recovery making this larynx preservation procedure more accessible to patients and surgeons.

Functional outcomes suggest that the procedure is repeatable and sufficiently safe (93).

Aiming to further reduce the overall impact of surgery, Spriano et al. have recently proposed a limited lateral cervicotomy approach which preserves anterior healthy tissue with evident better aesthetic outcomes and probably improved functional outcomes as well (95, 96).

## Conclusion

Why is interest in partial laryngeal surgery growing again today? Single and multi-institutional series support OLOPS by OPHLs as a safe technique in the management of laryngeal cancer in carefully selected stages, having standardized high OS, LRC, and DFS as well as acceptable functional outcomes.

Similar outcomes, although worse functionally, can be achieved in radio-recurrent or TLM-recurrent patients, determining inclusion of these procedures as a salvage option in selected patients.

Great variability is still observed in functional results, making research seeking a wide consensus on rehabilitation after OPHLs the field most likely to witness extensive improvements for the patient.

## Author Contributions

EC: surgeon who performed study conception and design, drafting the article, final approval of the version to be published. GS: surgeon who performed the final approval of the version to be published.

### Conflict of Interest Statement

The authors declare that the research was conducted in the absence of any commercial or financial relationships that could be construed as a potential conflict of interest.

## Abbreviations

MPT: maximum phonation time
OLOPS: open laryngeal organ preservation surgery
OPHL: open partial horizontal laryngectomy
PEG: percutaneous endoscopic gastrostomy
SCPL: supracricoid partial laryngectomy
STPL: supratracheal partial laryngectomy
TL: total laryngectomy
TLM: transoral laser microsurgery
TORS: transoral robotic surgery.
